# New investigation of bats-hosts-reservoir-people coronavirus model and application to 2019-nCoV system

**DOI:** 10.1186/s13662-020-02831-6

**Published:** 2020-08-03

**Authors:** Wei Gao, Haci Mehmet Baskonus, Li Shi

**Affiliations:** 1 School of Information Science and Technology, Yunnan Normal University, Yunnan, China; 2 Department of Mathematics and Science Education, Harran University, Sanliurfa, Turkey; 3 Institute of Medical Biology, Chinese Academy of Medical Sciences & Peking Union Medical College, Kunming, 650092 China

**Keywords:** 2019 novel coronavirus (2019-nCoV), Variational iteration method, Numerical solutions

## Abstract

According to the report presented by the World Health Organization, a new member of viruses, namely, coronavirus, shortly 2019-nCoV, which arised in Wuhan, China, on January 7, 2020, has been introduced to the literature. The main aim of this paper is investigating and finding the optimal values for better understanding the mathematical model of the transfer of 2019-nCoV from the reservoir to people. This model, named Bats-Hosts-Reservoir-People coronavirus (BHRPC) model, is based on bats as essential animal beings. By using a powerful numerical method we obtain simulations of its spreading under suitably chosen parameters. Whereas the obtained results show the effectiveness of the theoretical method considered for the governing system, the results also present much light on the dynamic behavior of the Bats-Hosts-Reservoir-People transmission network coronavirus model.

## Introduction

Today the whole world has been witnessing and suffering from a big pandemic disease, the novel coronavirus pneumonia, named as “2019 novel coronavirus (2019-nCoV)” [1]. The first wave of outbreak happened in Wuhan, China, and then quickly spread into some other parts of China and even to other countries due to its high person-to-person infection rate. The evidence of human-to-human transmission of it was identified by the National Health Commission (NHC) of the People’s Republic of China [2]. Based on statistics, there are more than 80,000 infected confirmed patients in China and around 7000 reported cases out of China (by the date March 16, 2020). Until now, around 150 countries reported the confirmed infection cases. In other words, it has been a global serious infective disease. Several researchers compared 2019-nCoV with SARS (severe acute respiratory syndrome) epidemic, which was out-broken in China in 2003 and pointed that the basic reproduction number (R0) of 2019-nCoV is estimated to be even higher than SARS with its reproduction number 6.47 vs SARS’s 4.91, which indicates a high spread and infection of 2019-nCoV and also how severe and acute the disaster we are confronted.

On December 2019, the symptom of 2019-nCoV infected patients was identified as fever, cough, breathing difficulties, and some other. Due to the long incubation period and mild symptoms, the suspicious infected people need to be observed for around 14 days. To reduce population flow and restrict the spread of the virus, corresponding virus dissemination control policies and relevant actions are being carried out at different levels. On January 23, Wuhan was locked down by strict restrictions of transport, and soon some other provinces announced to lockdown as well [3]. Chinese people were suggested to stay at home and avoid gathering, assembling, celebrations, visiting, and so on to reduce the virus dissemination. People were required to wear respirators in public areas. Some researchers pointed out some effective ways to control the spread of infectious virus, including school closure, case isolation, household quarantine, internal travel restrictions, and border control, which were proved to be helpful for the delay or reduction of virus infections [4–6]. Almost all the economic activities in some countries have been paused, which caused countless damages to humans’ lives, development, and also a large amount of financial pressure on these countries. Globally, Australia and New Zealand firstly released regulations to ban travellers who had been to China in the past 14 days. From December 2019 up to now, 2019-nCoV virus suddenly out-broke in other countries in flood. Italy, Japan, South Korea, Spain, and other countries were in the clouds of the virus as well, and some of them started to lockdown cities, restrict transports, close schools, and so on. The whole world is in damage by the 2019-nCoV and in the combat against coronavirus. Some other researchers devoted themselves to the drug and vaccine developments, however, there is no way to effectively eliminate the virus in the human’s body.

A large number of researchers have studied the 2019-nCoV from a wide range of perspectives, including diverse infectious diseases, microbiology, virology, respiratory system, biochemistry molecular biology, immunology, public environmental occupational health, genetics heredity, veterinary sciences, environmental sciences ecology, and pathology. Most of them are conducted by the USA and China, followed by Saudi Arabia, South Korea, and Germany. According to Tian [7] and others, the differences of RBD between SARS-CoV and 2019-nCoV is of significance for the cross-reactivity of neutralizing antibodies, and a SARS-CoV-specific human monoclonal antibody CR3022 could bind potently with 2019-nCoV RBD (KD of 6.3 nM). As for the origins of 2019-nCoV virus, 2019-nCoV, Benvenuto et al. [8] held that 2019nCoV could be considered a coronavirus distinct from SARS virus, probably transmitted from bats or another host, where mutations conferred upon it the ability to infect humans, and they also proposed a preliminary evolutionary and molecular epidemiological analysis of this new virus, considering high genetic similarity between 2019-nCoV and the Severe Acute Respiratory Syndrome coronavirus (SARS-CoV), and leveraging existing immunological studies of SARS-CoV, Ahmed, Quadeer, and McKay [9] devoted themselves to seeking for gaining insights for vaccine design against 2019-nCoV.

With the increasing of virus spread and the ongoing related research both at domestic and international, one question still hinders human’s knowledge of the 2019-nCoV: What is the original source of such a virus and how can it transmit to human. In this paper, we intend to study a mathematical model called the Bats-Hosts-Reservoir-People coronavirus (BHRPC) model for the transfer of 2019-nCoV from the reservoir to people. By using a powerful numerical method we gain its spreading simulations under suitably chosen parameters. The obtained results show the effectiveness of the theoretical method considering the governing system and also present much light on the dynamic behavior of the Bats-Hosts-Reservoir-People transmission network coronavirus model. In this regards, more recently, some experts have investigated some important nonlinear models arising in real-world problems [10–16].

One of such problems has been mathematically developed by Chen et al. for simulating the phase-based transmissibility of a novel coronavirus as 2019-nCoV [17] defined by

1 $$ \begin{gathered} \frac{du ( t )}{dt} = n_{p} - m_{p}u ( t ) - b_{p}u ( t ) \bigl[ y ( t ) + \kappa f ( t ) \bigr] - b_{w}u ( t )w ( t ), \\ \frac{dv ( t )}{dt} = b_{p}u ( t ) \bigl[ y ( t ) + \kappa f ( t ) \bigr] + b_{w}u ( t )w ( t ) - ( 1 - \delta_{p} ) \omega_{p}v ( t ) - \delta_{p}\omega '_{p}v ( t ) - m_{p}v ( t ), \\ \frac{dy ( t )}{dt} = ( 1 - \delta_{p} )\omega_{p}v ( t ) - ( \gamma_{p} + m_{p} )y ( t ), \\ \frac{df ( t )}{dt} = \delta_{p}\omega '_{p}v ( t ) - \bigl[ \gamma '_{p} + m_{p} \bigr]f ( t ), \\ \frac{dr ( t )}{dt} = \gamma_{p}y ( t ) + \gamma '_{p}f ( t ) - m_{p}r ( t ), \\ \frac{dw ( t )}{dt} = \varepsilon \bigl[ y ( t ) + cf ( t ) - w ( t ) \bigr], \end{gathered} $$

where $n_{p}$, $m_{p}$, $b_{p}$, *κ*, $b_{w}$, $\delta_{p}$, *ε*, *c* are real nonzero constants. The initial conditions for this system are shortly given by

2 $$ \begin{gathered} u(0)=\beta_{1},\qquad v(0)=\beta_{2},\qquad y(0)=\beta_{3},\qquad f(0)= \beta_{4},\qquad r(0)=\beta_{5}, \\ \omega(0)=\beta_{6},\qquad \omega_{p}'(0)=\beta_{7}, \qquad w(0)=\beta_{10},\qquad \gamma_{p}'(0)=\beta_{8},\qquad \gamma(0)= \beta_{9}.\end{gathered} $$

Equation (1) is used to describe the phase-based transmissibility of a novel coronavirus from source to people. In Eq. (1), *u* is the susceptible people, *v* is used to symbolize exposed people, *y* is the symptomatic infected people, *f* is asymptomatic infected people, *r* is removed people (recovered and died people), $n_{p}$ is the birth rate, $m_{p}$ is the death rate of people, *w* is the reservoir (the seafood area), $1 / \omega_{B}$ is the incubation period of bat infection, and $1 / \gamma_{B}$ is the infectious period of bat infection [17]. Khan et al. [10] have investigated the endemic equilibria, stability, and global sensitivity of system (1). If $r_{0} < 1$, then system (1) is locally asymptotically stable [10, 18], and the outbreak will fade away [18]. When $r_{0} > 1$, the outbreak will occur [18], and it is not stable. The used data for system (1) are for Wuhan, China, [17, 19]. Moreover, mathematical analysis and applications of dengue fever outbreak and epidemiology in the sense of fractional have been investigated [20, 21]. Recently, a numerical scheme based on the Newton polynomial has been applied successfully to observe important properties of the spread of COVID-19 with new fractal-fractional operators [22]. Some close relationships of COVID-19 with HIV have been presented in [23]. Many applications of fractional- or integer-order mathematical models explaining more detailed informations about the real-world problems have been presented in a detailed manner [24–50]. In this paper, we investigate the numerical distributions of 2019-nCoV according to time with the help of several approaching terms of VIM.

## Some important properties of VIM

In 1999, VIM, one of the most powerful numerical methods, was firstly developed by He [51–54] for numerical investigation and exceeds the difficulties of the perturbation or Adomian functions. Later, Wazwaz has applied VIM for investigating linear and nonlinear wave equations along with wave-like equations [55] and Laplace equation [56]. Moreover, many applications of VIM have been observed for various models [14–17, 51–60]. We consider differential equations of the form

3 $$ Lu + Nu = f ( x ), $$

where *L* and *N* are linear and nonlinear operators, respectively, [55], and $f ( x )$ is a source inhomogeneous term. According to basic concepts of VIM presented by He, we construct the following iteration formula for Eq. (3) [51–54]:

4 $$ u_{n + 1} ( x ) = u_{n} ( x ) + \int_{0}^{t} \lambda ( \tau ) \bigl[ L \widetilde{u}_{n} ( \tau ) + R\widetilde{u}_{n} ( \tau ) + N\widetilde{u}_{n} ( \tau ) - f ( \tau ) \bigr]\,\mathrm{d} \tau, $$

where the parameter *λ* is a general Lagrange multiplier, which can be optimally identified via the variational theory, the subscript *n* denotes the *n*th-order approximation, and $\widetilde{u}_{n}$ is considered as a restricted variation, which means $\delta \widetilde{u}_{n} = 0$. Clearly, the main steps of VIM first require the determination of the Lagrange multiplier *λ*, which needs to be optimally identified. Once *λ* is determined, the successive approximations $u_{n + 1}$, $n \ge 0$, of the solution *u* are obtained upon using a suitably selected function $u_{0}$, which satisfies the boundary conditions. Then the solution is given by

5 $$ u = \lim_{n \to \infty} u_{n}. $$

## Application of VIM to COVID-19 system

In this subsection, by using VIM we numerically investigate the Bats-Hosts-Reservoir-People coronavirus model. According to VIM iteration structure, we can write Eq. (1) in the following form:

6 $$ \begin{gathered} u_{k + 1} = u_{k} + \int_{0}^{t} \lambda \biggl[ \frac{du_{k}}{d\tau} - n_{p} + m_{p}u_{k} + b_{p}u_{k} [ y_{k} + \kappa f_{k} ] + b_{w}u_{k}w_{k} \biggr] \,d\tau, \\ v_{k + 1} = v_{k} + \int_{0}^{t} \lambda \biggl[ \frac{dv_{k}}{d\tau} - b_{p}u_{k} [ y_{k} + \kappa f_{k} ] - b_{w}u_{k}w_{k} \\ \phantom{v_{k + 1} =}{} + ( 1 - \delta_{p} )\omega_{p}v_{k} + \delta_{p}\omega '_{p}v_{k} - m_{p}v_{k} \biggr] \,d\tau, \\ y_{k + 1} = y_{k} + \int_{0}^{t} \lambda \biggl[ \frac{dy_{k}}{d\tau} - ( 1 - \delta_{p} )\omega_{p}v_{k} + ( \gamma_{p} + m_{p} )y_{k} \biggr] \,d \tau, \\ f_{k + 1} = f_{k} + \int_{0}^{t} \lambda \biggl[ \frac{df_{k}}{d\tau} - \delta_{p}\omega '_{p}v_{k} + \bigl[ \gamma '_{p} + m_{p} \bigr]f_{k} \biggr]\, d\tau, \\ r_{k + 1} = r_{k} + \int_{0}^{t} \lambda \biggl[ \frac{dr_{k}}{d\tau} - \gamma_{p}y_{k} - \gamma '_{p}f_{k} + m_{p}r_{k} \biggr] \,d\tau, \\ w_{k + 1} = w_{k} + \int_{0}^{t} \lambda \biggl[ \frac{dw_{k}}{d\tau} - \varepsilon y_{k} - \varepsilon cf_{k} + \varepsilon w_{k} \biggr]\, d\tau, \end{gathered} $$

where $k = 0,1,2,3, \dots$. It produces the stationary condition

7 $$ \lambda = - 1. $$

Substituting Eq. (7) into Eq. (6), we find the following iteration equation:

8 $$ \begin{gathered} u_{k + 1} = u_{k} - \int_{0}^{t} \biggl[ \frac{du_{k}}{d\tau} - n_{p} + m_{p}u_{k} + b_{p}u_{k} [ y_{k} + \kappa f_{k} ] + b_{w}u_{k}w_{k} \biggr]\, d\tau, \\ v_{k + 1} = v_{k} - \int_{0}^{t} \biggl[ \frac{dv_{k}}{d\tau} - b_{p}u_{k} [ y_{k} + \kappa f_{k} ] - b_{w}u_{k}w_{k} \\ \phantom{v_{k + 1} =}{} + ( 1 - \delta_{p} )\omega_{p}v_{k} + \delta_{p}\omega '_{p}v_{k} - m_{p}v_{k} \biggr] \,d\tau, \\ y_{k + 1} = y_{k} - \int_{0}^{t} \biggl[ \frac{dy_{k}}{d\tau} - ( 1 - \delta_{p} )\omega_{p}v_{k} + ( \gamma_{p} + m_{p} )y_{k} \biggr] \,d\tau, \\ f_{k + 1} = f_{k} - \int_{0}^{t} \biggl[ \frac{df_{k}}{d\tau} - \delta_{p}\omega '_{p}v_{k} + \bigl[ \gamma '_{p} + m_{p} \bigr]f_{k} \biggr] \,d\tau, \\ r_{k + 1} = r_{k} - \int_{0}^{t} \biggl[ \frac{dr_{k}}{d\tau} - \gamma_{p}y_{k} - \gamma '_{p}f_{k} + m_{p}r_{k} \biggr] \,d\tau, \\ w_{k + 1} = w_{k} - \int_{0}^{t} \biggl[ \frac{dw_{k}}{d\tau} - \varepsilon y_{k} - \varepsilon cf_{k} + \varepsilon w_{k} \biggr] \,d\tau. \end{gathered} $$

With the help of some computational software algorithm, by considering Eq. (2) with initial values we get the first approximate components of the system for $k=0$:

$$\begin{gathered} u_{1} = u_{0} - \int_{0}^{t} \biggl[ \frac{du_{0}}{d\tau} - n_{p} + m_{p}u_{0} + b_{p}u_{0} [ y_{0} + \kappa f_{0} ] + b_{w}u_{0}w_{0} \biggr] \,d\tau, \\ v_{1} = v_{0} - \int_{0}^{t} \biggl[ \frac{dv_{0}}{d\tau} - b_{p}u_{0} [ y_{0} + \kappa f_{0} ] - b_{w}u_{0}w_{0} \\ \phantom{v_{1} =}{}+ ( 1 - \delta_{p} )\omega_{p} ( 0 )v_{0} + \delta_{p}\omega '_{p} ( 0 )v_{0} - m_{p}v_{0} \biggr] \,d\tau, \\ y_{1} = y_{0} - \int_{0}^{t} \biggl[ \frac{dy_{0}}{d\tau} - ( 1 - \delta_{p} )\omega_{p} ( 0 )v_{0} + \bigl( \gamma_{p} ( 0 ) + m_{p} \bigr)y_{0} \biggr] \,d\tau, \\ f_{1} = f_{0} - \int_{0}^{t} \biggl[ \frac{df_{0}}{d\tau} - \delta_{p}\omega '_{p} ( 0 )v_{0} + \bigl[ \gamma '_{p} ( 0 ) + m_{p} \bigr]f_{0} \biggr] \,d\tau, \\ r_{1} = r_{0} - \int_{0}^{t} \biggl[ \frac{dr_{0}}{d\tau} - \gamma_{p} ( 0 )y_{0} - \gamma '_{p} ( 0 )f_{0} + m_{p}r_{0} \biggr] \,d\tau, \\ w_{1} = w_{0} - \int_{0}^{t} \biggl[ \frac{dw_{0}}{d\tau} - \varepsilon y_{0} - \varepsilon cf_{0} + \varepsilon w_{0} \biggr]\, d\tau. \end{gathered} $$

Thus we get the first approach of $u_{n}$, $v_{n}$, $y_{n}$, $f_{n}$, $r_{n}$, $w_{n}$ as follows:

9 $$\begin{aligned}& u_{1} ( t ) = \beta_{1} + \tau_{1}t, \\& v_{1} ( t ) = \beta_{2} + \tau_{2}t, \\& y_{1} ( t ) = \beta_{3} + \tau_{3}t, \\& f_{1} ( t ) = \beta_{4} + \tau_{4}t, \\& r_{1} ( t ) = \beta_{5} + \tau_{5}t, \\& w_{1} ( t ) = \beta_{10} + \tau_{6}t, \end{aligned}$$

where, for simplicity, we have taken

$$\begin{gathered} \tau_{1} = n_{p} - m_{p} \beta_{1} - b_{p}\beta_{1} \beta_{3} - \kappa b_{p}\beta_{1} \beta_{4} - b_{w}\beta_{1} \beta_{10}, \\ \tau_{2} = b_{p}\beta_{1} [ \beta_{3} + \kappa \beta_{4} ] + b_{w} \beta_{1}\beta_{10} - ( 1 - \delta_{p} ) \beta_{6}\beta_{2} - \delta_{p} \beta_{7}\beta_{2} + m_{p} \beta_{2}, \\ \tau_{3} = ( 1 - \delta_{p} )\beta_{6} \beta_{2} - ( \gamma_{0} + m_{p} ) \beta_{3}, \\ \tau_{4} = \delta_{p}\beta_{7} \beta_{2} - [ \beta_{8} + m_{p} ] \beta_{4}, \\ \tau_{5} = \gamma_{0}\beta_{3} + \gamma '_{k}\beta_{4} - m_{p}r_{0}, \\ \tau_{6} = \varepsilon \beta_{3} + \varepsilon c \beta_{4} - \varepsilon \beta_{10}. \end{gathered} $$

Now we obtain the second components of the variables $u_{n}$, $v_{n}$, $y_{n}$, $f_{n}$, $r_{n}$, $w_{n}$ for $k=1$:

$$\begin{gathered} u_{2} = u_{1} - \int_{0}^{t} \biggl[ \frac{du_{1}}{d\tau} - n_{p} + m_{p}u_{1} + b_{p}u_{1} [ y_{1} + \kappa f_{1} ] + b_{w}u_{1}w_{1} \biggr]\, d\tau, \\ v_{2} = v_{1} - \int_{0}^{t} \biggl[ \frac{dv_{1}}{d\tau} - b_{p}u_{1} [ y_{1} + \kappa f_{1} ] - b_{w}u_{1}w_{1}+ ( 1 - \delta_{p} )\omega_{p}v_{1} + \delta_{p}\omega '_{p}v_{1} - m_{p}v_{1} \biggr] \,d\tau, \\ y_{2} = y_{1} - \int_{0}^{t} \biggl[ \frac{dy_{1}}{d\tau} - ( 1 - \delta_{p} )\omega_{p}v_{1} + ( \gamma_{p} + m_{p} )y_{1} \biggr]\, d\tau, \\ f_{2} = f_{1} - \int_{0}^{t} \biggl[ \frac{df_{1}}{d\tau} - \delta_{p}\omega '_{p}v_{1} + \bigl[ \gamma '_{p} + m_{p} \bigr]f_{1} \biggr] \,d\tau, \\ r_{2} = r_{1} - \int_{0}^{t} \biggl[ \frac{dr_{1}}{d\tau} - \gamma_{p}y_{1} - \gamma '_{p}f_{1} + m_{p}r_{1} \biggr] \,d\tau, \\ w_{2} = w_{1} - \int_{0}^{t} \biggl[ \frac{dw_{1}}{d\tau} - \varepsilon y_{1} - \varepsilon cf_{1} + \varepsilon w_{1} \biggr]\, d\tau, \end{gathered} $$

which gives

10 $$ \begin{gathered} u_{2} ( t ) = \beta_{1} + ( \tau_{1} + \tau_{7} )t + \frac{\tau_{8}}{2}t^{2} + \frac{\tau_{9}}{3}t^{3}, \\ v_{2} ( t ) = \beta_{2} + ( \tau_{2} + \tau_{10} )t + \frac{\tau_{11}}{2}t^{2} + \frac{\tau_{12}}{3}t^{3}, \\ y_{2} ( t ) = \beta_{3} + ( \tau_{3} + \tau_{13} )t + \frac{\tau_{14}}{2}t^{2}, \\ f_{2} ( t ) = \beta_{4} + ( \tau_{4} + \tau_{15} )t + \frac{\tau_{16}}{2}t^{2}, \\ r_{2} ( t ) = \beta_{5} + ( \tau_{5} + \tau_{17} )t + \frac{\tau_{18}}{2}t^{2}, \\ w_{2} ( t ) = \beta_{10} + ( \tau_{6} + \tau_{19} )t + \frac{\tau_{20}}{2}t^{2}, \end{gathered} $$

where

$$\begin{gathered} \tau_{7} = \tau + n_{p} - m_{p}\beta_{1} - b_{p}\beta_{1} \beta_{3} - b_{p}\beta_{1}\kappa \beta_{4} - \beta_{1}\beta_{10}b_{w}, \\ \tau_{8} = m_{p}\tau_{1} + b_{p}\tau_{1}\beta_{3} + \tau_{3}b_{p}\beta_{1} + \tau_{6}b_{w}\beta_{1} + b_{p} \tau_{1}\kappa \beta_{4} + \tau_{4}b_{p} \beta_{1}\kappa + \tau_{1}\beta_{10}b_{w}, \\ \tau_{9} = - \tau_{6}b_{w} \tau_{1}t^{2} - \tau_{3}b_{p} \tau_{1}t^{2} - \tau_{4}b_{p} \tau_{1}\kappa, \\ \tau_{10} = \tau_{2} - \beta_{2} ( \omega_{p} - \omega_{p}\delta_{p} ) + b_{p}\beta_{1}\beta_{3} + b_{p} \kappa \beta_{1}\beta_{4} + b_{w} \beta_{10}\beta_{1} - \beta_{2} \delta_{p}\omega '_{p} + m_{p} \beta_{2}, \\ \tau_{11} = - b_{p}\tau_{1} \beta_{3} - \tau_{3}b_{p}\beta_{1} - b_{p}\kappa \tau_{1}\beta_{4} - \tau_{4}b_{p}\kappa \beta_{1} - \beta_{10}b_{w}\tau_{1} - \tau_{6}b_{w}\beta_{1} \\ \phantom{\tau_{11} =}{}+ \tau_{2} ( \omega_{p} - \omega_{p}\delta_{p} ) + \tau_{2} \delta_{p}\omega '_{p} - m_{p} \tau_{2}, \\ \tau_{12} = \tau_{3}b_{p} \tau_{1} + \tau_{4}b_{p}\kappa \tau_{1} + \tau_{6}b_{w} \tau_{1}, \\ \tau_{13} = \tau_{3} - \beta_{2} ( - 1 + \delta_{p} )\omega_{p} - \beta_{3} ( \gamma_{p} + m_{p} ), \\ \tau_{14} = + \tau_{2} ( - 1 + \delta_{p} ) \omega_{p} + \tau_{3} ( \gamma_{p} + m_{p} ), \\ \tau_{15} = \tau_{4} + \beta_{2} \delta_{p}\omega '_{p} - \beta_{4} \bigl( \gamma '_{p} + m_{p} \bigr), \\ \tau_{16} = \tau_{4} \bigl( \gamma '_{p} + m_{p} \bigr) - \tau_{2}\delta_{p}\omega '_{p}, \\ \tau_{17} = \tau_{5} + \beta_{3} \gamma_{p} + \gamma '_{p} \beta_{4} - \beta_{5}m_{p}, \\ \tau_{18} = - \gamma '_{p} \tau_{4} + \tau_{5}m_{p} - \tau_{3}\gamma_{p}, \\ \tau_{19} = \tau_{6} + \varepsilon \beta_{3} + \varepsilon c\beta_{4} - \varepsilon \beta_{10}, \\ \tau_{20} = - \varepsilon c\tau_{4} - \varepsilon \tau_{3} + \varepsilon \tau_{6}. \end{gathered} $$

**Figure 1:**
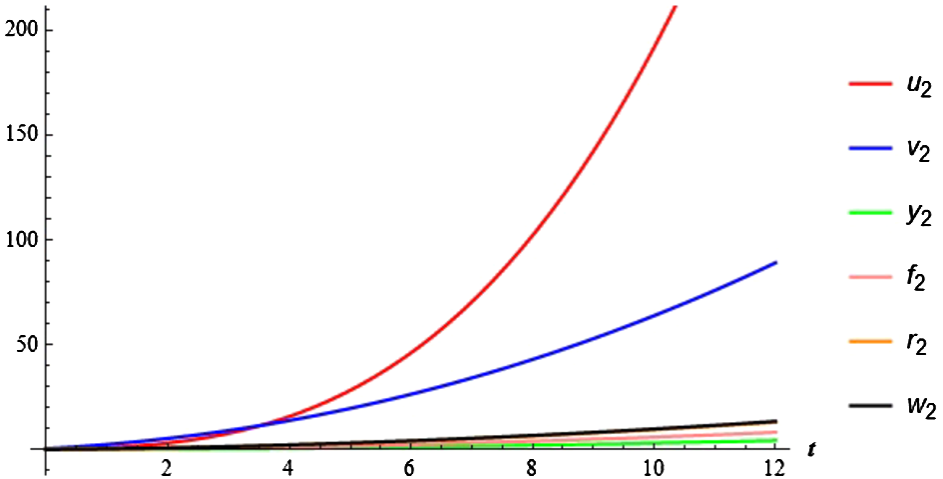
Two-dimensional surfaces of system (10)

The remaining components of the iteration formula (5) can be found in the same manner using a similar algorithm via various computational schemes. In this work, we observe the spreading rate of the Bats-Hosts-Reservoir-People coronavirus model from reservoir to people under suitably chosen values of the parameters, reported by experts in Wuhan area of China. We obtain two-dimensional simulations of the second terms $u_{2}$, $v_{2}$, $y_{2}$, $f_{2}$, $r_{2}$, $w_{2}$ of $u_{n}$, $v_{n}$, $y_{n}$, $f_{n}$, $r_{n}$, $w_{n}$ as in Fig. 1.

## Conclusions

**Figure 2:**
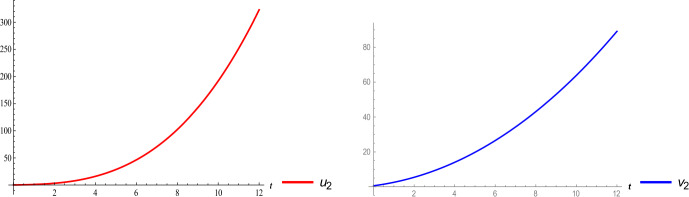
Two-dimensional surfaces of susceptible people and exposed people of system (10)

**Figure 3:**
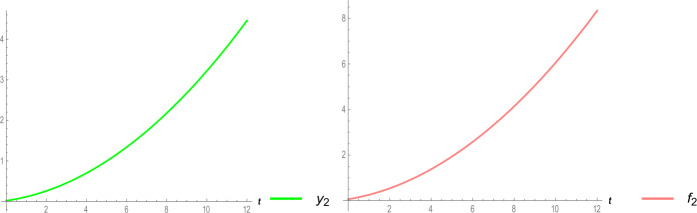
Two-dimensional surfaces of symptomatic infected people and asymptomatic infected people of system (10)

**Figure 4:**
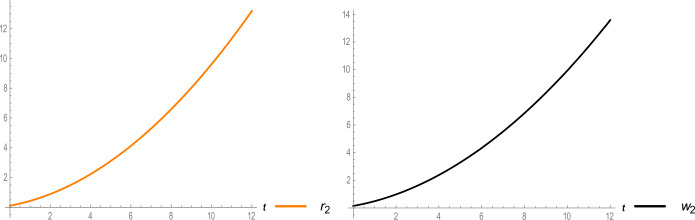
Two-dimensional surfaces of removed people (recovered or died people) and reservoir (the seafood area) of system (10)

In this paper, we have successfully applied VIM to numerical investigation of the 2019-nCoV model. This method is based on a series solution terms of iteration. Only in the second terms of iteration, we have obtained numerical results for system (10). Under suitably chosen values of the parameters, reported by WHO, we have plotted the numerical results. According to Figs. 1, 2, 3, 4, we observed that VIM produces similar distributions for susceptible people, which increase exponentially, when we compare the simulated data with Fig. 2 in [17]. Moreover, we can say that each class of system (1) has also simulated estimated behaviors from them. Furthermore, the spread of the 2019-nCoV with susceptible people $u_{2}$ is faster than the others, such as $v_{2}$, $y_{2}$, $f_{2}$, $r_{2}$, $w_{2}$. Finally, from the second terms of the proposed algorithm we can observe that susceptible people will affect more and more people from all over the world. As a future direction of this concept, application of powerful projected tools may be studied. These produce more comprehensive results on the mathematical system of 2019-nCoV.
